# Attention to the body! Comparing the connection between interoceptive abilities and somatic complaints of women with and without history of intimate partner violence

**DOI:** 10.1177/17455057251326013

**Published:** 2025-04-28

**Authors:** Joana Machorrinho, José Marmeleira, Graça Duarte Santos, Guida Veiga

**Affiliations:** 1 Comprehensive Health Research Center (CHRC), Universidade de Évora, Évora, Portugal; 2 Departamento de Desporto e Saúde, Escola de Saúde e Desenvolvimento Humano, Universidade de Évora, Évora, Portugal

**Keywords:** somatic complaints, Portugal, interoception, attention regulation, domestic violence

## Abstract

**Background:**

Somatic complaints are a critical burden to women, particularly to those women who survived intimate partner violence (IPV). The way women feel, perceive, and relate to their own body, that is, interoception, seems to have a significant role in the pathway to somatic complaints. However, to the best of our knowledge, no study has yet explored the relationship between interoception and somatic complaints of women survivors of IPV.

**Objectives:**

To deepen the understanding of the underlying interoceptive mechanisms of somatic complaints experienced by women survivors of IPV.

**Design:**

Cross-sectional study.

**Method:**

Women with (*N* = 44) and without (*N* = 52) history of IPV were assessed regarding interoceptive accuracy, interoceptive sensibility, and somatic complaints. Associations between both variables in each group were examined, and a hierarchical regression analysis assessed to what extent somatic complaints were explained by the interoceptive abilities, with the mediating role of IPV group membership.

**Results:**

Women survivors of IPV reported more somatic complaints (*p* < 0.001), which were negatively associated with interoceptive attention regulation. The opposite association was found in women who have never experienced IPV. For the IPV group, the interoceptive attention regulation, added to age and the index of the violence suffered, explains 43% of the variance in somatic complaints.

**Conclusion:**

The findings suggest that for women with history of IPV, but not for those without, the ability to regulate the attention given to bodily sensations is a mediator of women’ somatic complaint. Thereby we suggest that interoceptive attention regulation can be a promising therapeutic aim, for women recovering from IPV.

## Plain language summary

Regulating attention to bodily sensations contributes to decreasing the health complaints of women survivors of intimate partner violence

**Why was the study done?** Women who suffered violence in an intimate relationship report more health complaints than women who have never suffered such violence. In general, it is known that different ways of feeling the body distinctively influence how many and how intensively one’s report health complaints. It is also known that women who suffered violence have a different way of feeling the body and its sensations. However, how this way of feeling influences the health complaints of women survivors of intimate partner violence remains unexplored. **What did the researchers do?** The research team studied different abilities of feeling the body of women with (44) and without (52) history of intimate partner violence. Also, the health complaints of each were assessed, and how those were influenced by feeling the body was examined. What did the researchers find? Women survivors of intimate partner violence reported more difficulties falling asleep, migraines, panic attacks, anxiety and attention and memory struggles. Furthermore, for women who have experienced this type of violence—but not for those who have not—greater ability to regulate attention toward the body (i.e., focusing on the body only when necessary) was associated with fewer health complaints. **What do the findings mean?** The findings suggest that for women who suffered intimate partner violence, health care must promote the ability to regulate the attention given to the body, helping women to have better health, better emotional regulation, and lower risk of developing negative symptoms.

## Introduction

Somatic complaints are frequently reported by survivors of intimate partner violence (IPV).^1,2^ Complaints like back pain, headache, and dizziness generally derive from the distressing perception of bodily sensations, impairing daily functioning.^3^ Indeed, the so-called interoception, that is, the process of *sensing and perceiving internal bodily sensations, like heartbeat, breathing, or pain*, has been argued as a critical explanatory mechanism for the experience of somatic complaints.^4^ However, external factors have also a role in this pathway. For example, the experience of traumatic situations, such as IPV, may have an impact on interoceptive abilities. Understanding if and how this perception and interpretation of bodily signals exacerbates somatic complaints of IPV survivors is paramount for improving medical care and developing effective integrative therapeutic interventions.

For years, the evidence has pointed to somatic complaints arising from the ability to perceive bodily signals accurately—interoceptive accuracy; thus, bottom-up sensory information would originate and exacerbate the perception of a symptom.^5,6^ However, contradictory findings got this link questioned, suggesting that top-down cognitive and affective processes significantly alter this relationship and influence bodily signals’ perception, interpretation, and reporting.^3,4,7,8^

Heightened arousal is one of the main interferences in interoception.^9,10^ It reflects mainly on increased bodily signals,^9,11^ increased perception of minor bodily signals^4,12,13^ or on even minor bodily signals being categorized as threatening, pathological, or stressful.^10,13,14^ The resulting misinterpretation of actual physiological signals and their slight changes result in a cycle of hypervigilance toward the body that continuously exacerbates symptoms’ perception, thereby contributing to symptom maintenance.^3,15,16^ Simultaneously, dysfunctional behavioral processes—such as avoiding physical activities and withdrawing from beneficial social contacts—may contribute to distress and other somatic symptoms.^17^

Considering this, an approach to understanding somatic complaints as a more complex process involving both bottom-up and top-down processes arose. The concept of somatosensory amplification refers to the tendency to experience bodily sensations as intense, catastrophic, and disturbing,^4,12,13^ involving either bottom-up processes (bodily signals) and top-down processes (as body hypervigilance; focus on slight and weak body sensations, and cortical reactions to sensations). According to Wolters et al.,^13^ the brain generates predictions about internal bodily states based on incoming interoceptive information and adjusts these predictions accordingly. However, when there is a misinterpretation or misperception of interoceptive sensations, those predictions do not match with the sensory input, resulting in perceived somatic symptoms that do not correspond to physiological changes.^3,13^ Furthermore, this biased interoception leads to individuals affirming symptoms readily in ambiguous situations.^18^ Altogether, these processes contribute to exacerbating somatic complaints, resulting in health worries, expectations of symptoms, and amplified perception of external threats.^12,13^

Moreover, studies on somatization, the tendency to express emotional distress as somatic symptoms,^19^ suggest that maladaptive defense mechanisms such as suppression and displacement of negative emotions are associated with the formation of somatic symptoms, possibly through difficulties handling emotional stimuli that interfere with sensorial information interpretation.^20^ In this sense, showing an inflexible response (as opposed to a flexible adaptation) to repeated or severe threats to the body (as is the case of IPV) is suggested to compromise the accurate perception of bodily signals and resulting in interoceptive illusions, therefore contributing to somatic complaints.^21^ Somatization is often found in people exposed to traumatic experiences, in particular in those experiencing posttraumatic stress disorder (PTSD)^20^ which, in turn, is based on a condition of dysregulated arousal.^22^

Taken together, these findings contribute to the current efforts to conceptualize the different Posttraumatic Orientations to Bodily Signals, an umbrella term that encompasses the tendency to interpret bodily signals as catastrophic and frightful after exposure to trauma.^23,24^ However, in the literature review that supports this term, only 8% of the included studies were on interpersonal trauma or IPV,^25^ which highlights the need of more studies.

## The case of IPV

Women survivors of IPV often report medically unexplained symptoms in gastrointestinal, cardiopulmonary, neurological, sexual, and reproductive dimensions.^26,27^ Such symptoms have been reported by adults who suffered violence in childhood,^28^ adolescence,^29^ and adulthood,^30,31^ corroborating the long-lasting detrimental effects of interpersonal violence. Furthermore, the more violence a woman is exposed to, the higher the number of somatic symptoms reported,^1^ especially if they suffered sexual violence or abuse and if the abuse came from someone close to them.^32^

Trauma consequences, such as maladaptive defense mechanisms, altered brain connectivity, hypervigilance, dissociation, and psychopathology, contribute to either heightened or reduced alertness, a misinterpretation of interoceptive sensations, and somatic symptoms reporting.^16,33–35^ Also, bodily dissociation (i.e., the feeling of being separate from the body and with difficulty expressing emotions),^36^ another consequence of IPV,^31,35^ has already proven to be a significant mediator between traumatic experiences and emotion dysregulation.^37^ Bodily dissociation decreases the ability to be aware of bodily sensations, sustain attention toward the body, and trust in interoceptive sensations,^38^ thus adding as a possible mechanism linking interoception to somatic complaints in violence-related trauma.^16^

So far, research has shown that women survivors of IPV show a high report of somatic complaints, a low interoceptive accuracy, and difficulties in interoceptive self-regulation and in trusting in bodily sensations which relate to increased symptoms of PTSD, anxiety and depression.^31,39^ Despite these significant findings, the dynamics between interoception and somatic complaints of survivors of IPV remain unexplored. Moreover, considering that the cumulative aspect of IPV (co-occurrence of various forms of violence) has been related to the experience somatic symptoms of women exposed to violence across the lifespan, it is necessary to account for a violence index in related research.^40,41^ Hence, there is a need of a deeper understanding of the interplay between violence severity and interoceptive abilities and their role on somatic symptoms of IPV survivors.

## The present study

Somatic complaints have a heavy impact on daily functioning. Regarding survivors of IPV, the experience of somatic complaints increases if they experience more than one form of violence.^41^ One of the most acknowledged mechanisms for perceiving and reporting somatic complaints is interoception, namely interoceptive accuracy and interoceptive sensibility. As detailed in the sections above, biased interoception (e.g., high sensitivity to internal sensations combined with poor accuracy or misinterpretation of such signals) was both found in people with somatic symptom disorders^13^ and with violence-related trauma.^16,33,42^ However, the contribution of interoceptive abilities for somatic complaints of survivors of IPV still lacks research.

Therefore, the main aim of this study is to gain a deeper understanding of the relationship between somatic complaints and interoceptive abilities (accuracy and sensibility) of women survivors of IPV, accounting factors that knowingly contribute to both somatic complaints and/or interoception, such as age,^43^ mental health,^3,33,39^ and the violence suffered.^41^

To attain the aim of this study, the research strategy was threefold. First, we examined the somatic complaints, interoceptive abilities, violence index, and mental health (PTSD, depression and anxiety diagnoses) of women survivors of IPV, and compared them with women who have not experienced IPV. Based on previous research,^26,30,31,35,38^ we hypothesized that survivors of IPV show higher rates of somatic complaints, and a higher tendency for not-distracting themselves from negative sensations, as well as lower interoceptive accuracy, attention regulation, self-regulation, and trusting in bodily sensations (H1). In parallel, we expected a higher violence index, and a higher report of mental health diagnoses.^1,16,31,35^

Second, we examined how somatic complaints were related with interoceptive abilities and violence index, for women survivors of IPV and for women with no IPV history. Considering that age,^43^ mental health,^3,33,39^ and the history of violence^41^ influences both somatic complaints and/or interoception, these factors were accounted. Considering previous research, we hypothesized a positive association between Violence Index and somatic complaints (H2), and a negative association between somatic complaints and interoceptive accuracy,^39^ attention regulation, self-regulation, and trusting for women survivors of IPV (H3).^31,38^ However, for women non-victims of IPV, we hypothesized a negative association of somatic complaints with interoceptive accuracy, and the noticing, not-worrying, emotional awareness, and self-regulation scales of interoceptive sensibility (H4).^5,44,45^

Third, we investigated to what extent somatic complaints were explained by the interoception abilities correlated in the previous step, with the mediating role of IPV group membership. Although no previous study has examined this, we hypothesized that IPV group would play a mediating role in the relationship between somatic complaints and interoception, possibly in the accuracy, attention regulation, and trusting abilities (H5), since preliminary evidence suggests they behave differently for people with and without a history of IPV.

## Methods

This was a comparative study, for which the STROBE guidelines for cross-sectional studies were followed when preparing this manuscript.^47^

### Participants and procedure

OpenEpi was used to calculate the minimum required sample size for unmatched case–control studies.^48^ For this calculation, significance level (alpha) was set at 0.05, power at 90%, proportion of controls with exposure at 30%, ratio of sample size at 1, and hypothetical proportion of cases with exposure at 80%.^49^ Results indicated that a minimum of 23 women survivors of IPV and 23 women without experience of IPV were required. A total of 96 women (18–68 years old) participated in this study (Table 1). Of this, 44 were women survivors of IPV living in shelter homes (IPV group; 18–68 years old), and 52 were women recruited from the community who reported never suffering IPV (no-IPV group; 19–64 years old).

**Table 1.** Sample characteristics and descriptives.

Variables	Group IPV (*N* = 44)	Group no-IPV (*N* = 52)	*p*-value
	Mean (SD)	Mean (SD)	
Age	40.3 (11.2)	43.1 (12.5)	0.254
Mental health diagnosis			
PTSD, *n* (%)	6 (11.5)	0 (0.0)	0.021
Anxiety, *n* (%)	15 (28.8)	10 (22.7)	0.501
Depression, *n* (%)	32 (61.5)	12 (27.3)	<0.001
Violence index	2.9 (1.0)	0.3 (0.5)	<0.001
Somatic complaints	0.36 (0.27)	0.18 (0.18)	<0.001
Sleep problems	0.65 (0.48)	0.34 (0.48)	0.002
Panic attacks	0.21 (0.41)	0.02 (0.15)	0.006
Anxiety attacks	0.46 (0.50)	0.25 (0.44)	0.033
Respiratory problems	0.19 (0.40)	0.11 (0.32)	0.295
Auto-immune diseases	0.10 (0.30)	0.09 (0.29)	0.936
Chronic pain	0.29 (0.46)	0.16 (0.37)	0.136
Migraines	0.44 (0.50)	0.21 (0.41)	0.014
Attention and memory difficulties	0.42 (0.49)	0.23 (0.42)	0.044
Gastrointestinal problems	0.33 (0.59)	0.16 (0.48)	0.070
Cardiac/vascular problems	0.27 (0.49)	0.16 (0.37)	0.264

PTSD: posttraumatic stress disorder; IPV: intimate partner violence; SD: standard deviation.

The inclusion criteria were (a) being female, (b) aged ⩾18 years, and (c) having suffered IPV (for the IPV group) or (d) not having suffered IPV (for the no-IPV group). Women under 18 and/or with significant cognitive impairments that could affect comprehension of the assessments were excluded of this study.

For the IPV group, the study was presented to four shelter homes from the central and southern regions of Portugal. After approval from their managing entities, women were asked to participate, and a total of 47 accepted to enroll. Of these, 44 completed the assessments and were included in the analysis.

For the no-IPV group, a presentation of the study with an invitation to participate was disseminated using social media and the university communication channels, through a snowball effect. Of the 53 women from the local community who showed interest in participating, 52 completed the assessments and were included in the analysis.

Confidentiality was maintained and participation was subject to informed consent. Each testing session took about 45 min and was performed individually in a quiet room of the shelter—for the IPV group—or at university facilities or participants’ home—for the no-IPV group. After a brief explanation of the purpose of the study, participants filled out a sociodemographic survey assessing sociodemographic data, age, mental health diagnosis, medication, somatic complaints and forms (psychological, physical, and sexual), and contexts (intimate relationship, family relationship, or work relationship) of the violence suffered. Data were collected between November 2021 and January 2023.

This study followed trauma-informed and trauma-sensitive practices, namely through a researcher experienced in therapy, in establishing physical and relational safety and trust in the assessment setting, and in knowing how to avoid re-traumatization in such context.

### Instruments

Somatic complaints were assessed through a checklist of 10 somatic symptoms developed following previous conceptual frameworks.^50,51^ Women reported which complaints they experienced (e.g., sleep difficulties, migraines, chronic pain) in the past week. The total score was obtained by the mean of all the reported symptoms, with higher scores indicating more symptoms. In the present study, the Cronbach’s alpha of this instrument was 0.723.

Interoception was evaluated in two dimensions: interoceptive accuracy and interoceptive sensibility. For assessing interoceptive accuracy, we administered the heartbeat counting task.^52^ In a sitting position, participants were asked to count the heartbeats felt during each trial of 45, 35, 55, and 25 s, presented in a fixed order. A pulse oximeter was fitted on the index finger of their left hand for physiological heartbeat detection. For each trial, an accuracy score was derived: 1 − (real beats − reported beats)/((real beats + reported beats)/2). Resulting accuracy scores were averaged over the four trials, yielding an average value for each participant. Interoceptive sensibility was assessed through the self-report questionnaire multidimensional assessment of interoceptive awareness (MAIA).^53^ The Portuguese version of MAIA consists of 33 items and uses a 6-point Likert scale (0: never; 5: always), where higher scores indicate greater positive interoceptive sensibility. It evaluates seven dimensions: noticing (i.e., awareness of body sensations), not-distracting (i.e., tendency not to distract oneself from sensations of pain or discomfort), not-worrying (i.e., tendency not to worry with sensations of pain or discomfort), attention regulation (ability to sustain and control attention), emotional awareness (i.e., awareness of the connection between body sensations and emotional states), self-regulation (i.e., ability to regulate distress by attention to body sensations), and trusting (i.e., experience of one’s body as safe and trustworthy).^38,52^ In this study, the not-worrying scale showed a Cronbach’s alpha of 0.497; therefore, it was removed from the analysis. For all the other scales, Cronbach’s alphas ranged from 0.701 (noticing) to 0.876 (attention regulation).

Violence index was obtained by computing the sum of all the forms of violence suffered (psychological, physical, and sexual) ranging from 0 (zero) to 3 (three).

### Statistical analysis

The analysis plan followed the three steps and hypothesis testing mentioned in the “The Present Study” section.

Primarily, descriptive statistics of sociodemographic data, somatic symptoms, mental health medical diagnosis, violence history, and interoception skills were performed for both IPV and no-IPV groups. The Shapiro–Wilk test was used to check data normality. Reliability of the somatic complaints checklist and of each subscale of MAIA was checked through Cronbach’s alpha and inter-item correlation (Table 2). Independent sample *t*-tests (reporting *t*-test statistics, degrees of freedom, and *p*-values) were used to compare age, somatic complaints, mental health, and interoception between women with and without IPV history.

**Table 2.** Psychometric properties and mean scores (standard deviations) of the measures.

Variables	*N* items	Scale range	Cronbach’s α	Mean (SD)		*U*	*p*
				IPV	No-IPV		
Somatic complaints checklist	10	0.0–1.0	.723	0.4 (0.3)	0.2 (0.2)	691	<0.001
Interoceptive accuracy, HCT	1	0.0–0.9		0.4 (0.3)	0.4 (0.3)	1014	0.625
Interoceptive sensibility, MAIA							
Noticing	3	0.0–5.0	.701	3.8 (1.1)	3.8 (1.1)	1124	0.882
Not-distracting	4	0.0–5.0	.806	1.4 (1.1)	1.6 (1.1)	1068	0.578
Not-worrying	4	0.0–5.0	.497	2.6 (1.0)	2.5 (0.8)	1052	0.496
Attention regulation	7	0.4–5.0	.876	3.3 (1.2)	3.2 (0.8)	1099	0.743
Emotional awareness	5	0.2–5.0	.871	4.3 (0.8)	4.1 (0.9)	955	0.160
Self-regulation	7	0.0–5.0	.863	3.0 (1.2)	3.2 (0.8)	1080	0.640
Trusting	3	0.3–5.0	.857	3.1 (1.5)	3.8 (1.0)	904	0.076

HCT: heartbeat counting task; MAIA: multidimensional assessment of interoceptive awareness; IPV: intimate partner violence; SD: standard deviation.

Second, for each group, Spearman’s rho for non-normal variables was used to check partial correlations between somatic complaints, violence index, and interoceptive abilities, controlling for age, PTSD, and depression diagnosis, since those are known confounders for interoception variability and/or somatic complaints.

Third, hierarchical regression analysis was used to assess the moderating role of interoceptive abilities and violence index on somatic complaints (model 1), adding an interaction term between each factor and the group (model 2), to assess IPV-group membership mediator role. Adjusted *R*-squared terms were calculated to assess the proportion of variation in somatic symptoms predicted by the statistical models. Normality, linearity, and heteroscedasticity checks performed on the data confirmed that all model assumptions were met.

### Data cleaning

Few values (<5%) were missing, and Little’s test of missing completely at random (Little’s MCAR test; *p* > 0.05) indicated that these were missing at random. Therefore, all participants were included, and missing values were replaced by the mean value of the respective item scores. All statistical analyses were conducted using version 24.0 of SPSS (IBM Corp., Armonk, NY, USA, 2017) and significance level was set at *p* < 0.05 (two-tailed).

## Results

### Sociodemographics, mental health, somatic complaints, violence index, and interoceptive abilities of women with and without history of IPV

A total of 96 women participated in this study. Table 1 presents the characteristics of both IPV (*n* = 44) and no-IPV (*n* = 52) groups. The majority of IPV victims had a secondary educational level or less, whereas most of the non-victims had an educational level above the secondary level (*p* = 0.001). When compared to non-victims, IPV victims showed significantly higher frequency of PTSD and depression diagnosis, along with an increased violence index (*p* < 0.001) and report of somatic complaints (*p* < 0.001), namely sleep problems (*p* = 0.002), panic attacks (*p* = 0.006), anxiety attacks (*p* = 0.033), migraines (*p* = 0.014), and attention and memory difficulties (*p* = 0.044). IPV and no-IPV groups did not differ in age, interoceptive accuracy, or interoceptive sensibility.

### Correlations between somatic complaints and interoceptive abilities and violence index of women with and without history of IPV

Results from the second step showed that, for the IPV group, higher rates of somatic symptoms correlate with higher violence index (*p* < 0.001) and with lower attention regulation (*p* = 0.006), that is, lower ability to regulate the attention given to internal sensations. On the other hand, no significant correlations with somatic complaints were found for the no-IPV group (see Table 3).

**Table 3.** Correlations of violence and interoception with somatic complaints.

Variables	Somatic complaints	
	Group IPV (*N* = 44)	Group no-IPV (*N* = 52)
	Rho	Rho
Violence index	0.418*	0.126
IAc	0.173	−0.157
Noticing	−0.026	0.232
Not-distracting	−0.102	−0.050
Not-worrying	−0.217	0.028
Attention regulation	−0.295*	0.019
Emotional awareness	0.154	0.117
Self-regulation	−0.118	−0.035
Trusting	−0.041	−0.197

Controlled for age, PTSD, anxiety, and depression. IAc: interoceptive accuracy; IPV: intimate partner violence; PTSD: posttraumatic stress disorder; *p-value < .05.

### Examining the mediator role IPV-group membership in the contribution of interoceptive abilities to somatic complaints

In the final analysis, a hierarchical linear regression was used including age, violence index, and attention regulation as possible moderators of somatic complaints, adding an interaction term with group membership. Results (see Table 4) showed that when group interaction was added, interoceptive attention regulation explained the variations in somatic complaints of women survivors of IPV, above and beyond the contribution of age and of cumulative violence. This model explains 43% of the variance in somatic complaints. In the IPV group, interoceptive attention regulation is negatively associated with somatic complaints, while the opposite happens in the no-IPV group.

**Table 4.** Hierarchical regression analyses for interoception on somatic complaints.

Variable	Somatic complaints		
	*B*	*p*	95% CI
Step 1	*R*^2^ = 0.378, *p* = <0.001		
Age	0.386	<0.001	0.005, 0.012
Violence index	0.522	<0.001	0.058, 0.111
Attention regulation	−0.162	0.060	−0.079, 0.002
Step 2	∆*R*^2^ = 0.051, *p* = 0.053		
Age	0.253	0.018	0.001, 0.010
Violence index	0.460	0.264	−0.057, 0.206
Attention regulation	0.137	0.314	−0.031, 0.097
Group × age	0.485	0.066	0.000, 0.011
Group × violence index	0.233	0.614	−0.104, 0.175
Group × attention regulation	−0.765	0.007	−0.175, −0.029

CI: confidence interval.

**Figure 1.**
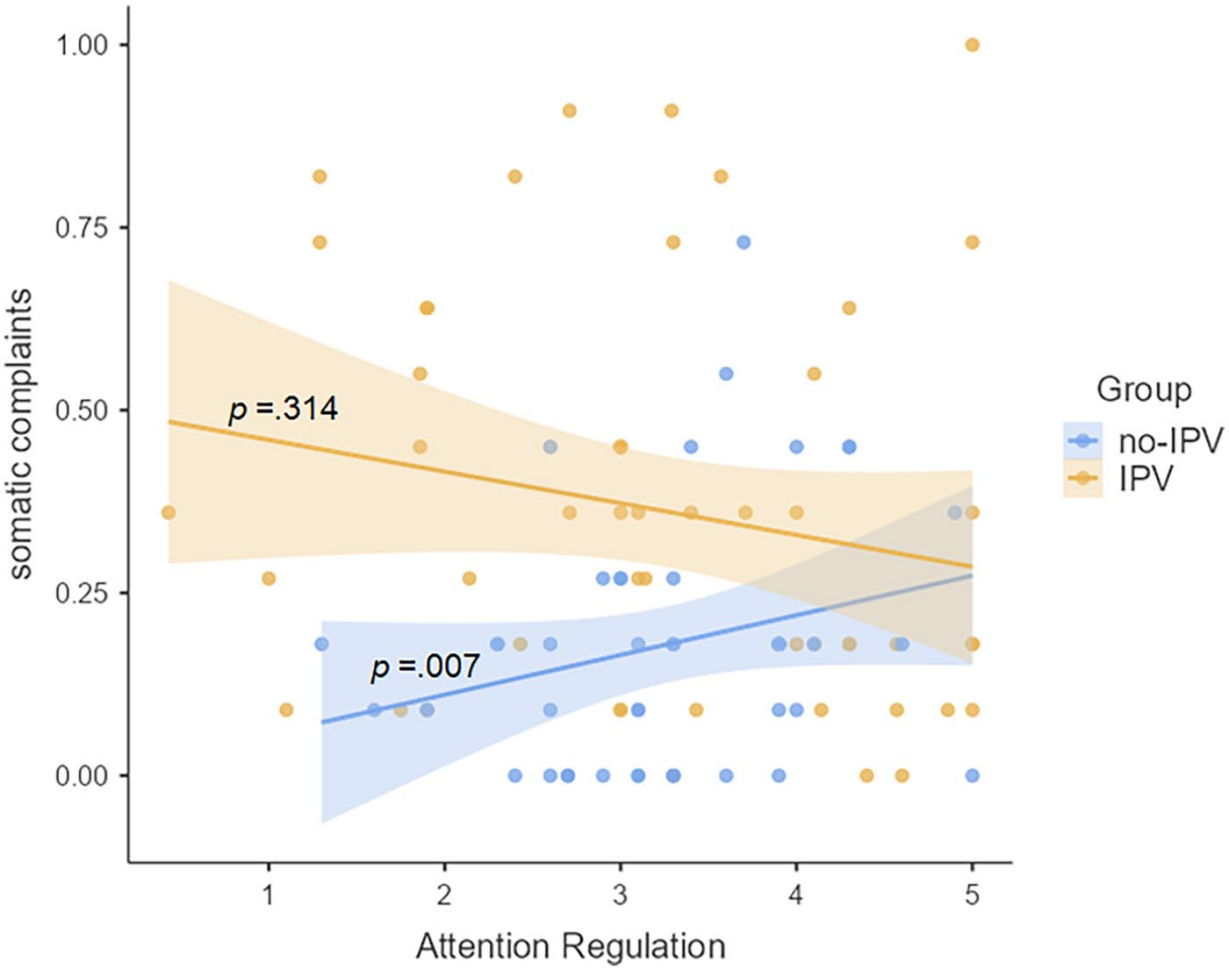
Scatterplot of the relationship between somatic complaints and interoceptive attention regulation, *per* group.

The scatterplots (Figure 1) confirm the contribution of interoceptive attention regulation to somatic complaints behave differently for women with and without IPV history, unlike the other interoceptive abilities assessed.

## Discussion

Somatic complaints are a critical burden to women, and particularly to those women who survived IPV. The way women feel, perceive, and relate to their own body seems to have a significant role in the pathway to somatic complaints. The current study examined the relationship between somatic complaints and interoceptive abilities of women with and without experience of IPV, in order to deepen our understanding of the underlying mechanisms of the experience of somatic complaints within this population. Indeed, our study showed that women survivors of IPV experience more somatic complaints than those without IPV, and that, above and beyond age and cumulative violence, interoceptive attention regulation plays a significant role in the experience of somatic complaints by women survivors of IPV.

The present study showed that IPV survivors were more likely to have a mental health diagnosis. In particular, 11.5% of these women had PTSD, and 61.5% had a diagnosis of depression, twice as much as the women who did not have a IPV history, which is in line with other studies.^35,54^ Although the prevalence of anxiety disorder was slightly higher (29%) for IPV survivors, than for women with no IPV history (23%), these differences were not statistically significant. Moreover, both frequencies were higher than a previous report on Portuguese adults’ mental health (21%).^55^ Nevertheless, survivors reported more anxiety symptoms and panic attacks than women with no IPV history. Altogether, these findings might suggest that women who suffer from IPV have fewer opportunities to access medical care.^56,57^

In general, as hypothesized (H1), women survivors of IPV reported more somatic complaints than women who have never suffered IPV.^1,26,30^ Besides, the experience of somatic complaints was related to a higher violence index, supporting Hypothesis 2. This finding strengthens the theory of exposure–response association between violence and symptoms, and reinforces the idea that somatic symptoms can be interpreted as markers of victimization.^17,29^

It is important to note that IPV survivors reported more somatic-cognitive complaints, that is, memory and attention difficulties, than non-victims of IPV (H1). These findings are consistent with previous studies^58,59^ and give support to Negrón-Oyarzo et al.’s^60^ hypothesis that those somatic-cognitive symptoms can be a consequence of IPV-related phenomena such as distress^60^ or PTSD,^61^ showing the impact of IPV on neuropsychological functioning.^58^ Alternatively, those somatic-cognitive complaints might be an indicator of attention-deficit and hyperactivity disorder (ADHD).^62^ It has been suggested a co-occurrence of genetic risks for both ADHD and severe IPV, either by parental mental illness leading to adverse childhood experiences that increase the risk of mental illness and IPV in adulthood, or by inherited genetic risk for mental illness increasing the likelihood of experiencing risk factors for IPV victimization.^63^ Therefore, symptoms of attention and memory difficulties are currently understood as either a risk factor or a sequelae of IPV victimization,^64–67^ and can be a crucial pathway to understand the relationship between interoception and somatic complaints in survivors of violence.

Considering the somatic complaints most frequently reported by women survivors of IPV, namely panic and anxiety attacks, attention and memory impairments, sleep difficulties and migraines, those fall under a symptomatic framework of heightened arousal and hypervigilance.^13,68,69^ Moreover, the prevalence of PTSD diagnosis among the IPV group supports this framework and is consistent with IPV-PTSD subjects showing increased activation and decreased connectivity among the anterior insula and the amygdala, expressing a disconnection among affective and limbic sensory systems, and an exaggerated sensitivity to contextual cues.^70^ These findings strengthen the ongoing conceptualization of a posttraumatic orientation to bodily signals^23^ and reinforce the need of studies specifically focused on the IPV-PTSD cluster. For instance, our findings partially support Hypothesis 3, as they show that, for women survivors of IPV, somatic complaints are negatively associated with interoceptive attention regulation, self-regulation and trusting in bodily sensations, but not with interoceptive accuracy. However, for women who never experienced IPV, the hypothesized negative correlation was only confirmed between somatic complaints, interoceptive accuracy and self-regulation, which were not statistically significative, therefore rejecting Hypothesis 4.

The influence of interoceptive attention regulation on somatic complaints differs for women based on their history of IPV. Our findings indicate that interoceptive attention regulation explains somatic complaints in women with a history of IPV, but not in those without, supporting Hypothesis 5. This sheds light on the varying impact of interoceptive bias on health within different relational contexts. Indeed, previous research showed that for populations with no history of violence, interoception (accuracy and sensibility) is not related to the experience of somatic complaints,^71^ which reinforces the idea that healthy individuals living in healthy environments and situations do not need to consciously focus on their bodies or divert their attention from external surroundings.^72^ In a somatic and emotionally healthy state, attention can be regulated to detect and interpret signals from the body only when needed, since it is generally focused on the external world.^73^ However, when external relational cues are frequently threatening and the body’s integrity or identity is at risk, one’s attention must constantly shift between cues to ensure survival. Hence, the negative impact of unpredictability on the experience of bodily sensations might increase the interoceptive bias and emotional distress.^3,74^

Concerning women survivors of IPV, the current study showed that interoceptive attention regulation plays a significant role in the experience of somatic complaints by women survivors of IPV. These findings suggest that for women who have lived in a violent relationship, it is of paramount importance to be able to sustain and control attention to body sensations. An adaptive attention regulation ability can help survivors to either divert attention from the distressing surroundings (preventing them from being continuously misinterpreted as dangerous) or use bodily sensations to self-regulate and manage health and illness symptoms adaptively.^74–77^ Indeed, such interoceptive ability has been shown to improve emotional and physiological regulation.^76^ Thereby, therapeutic interventions that focus on interoceptive attention regulation abilities can allow women survivors of IPV an inner distancing from ruminating negative and catastrophic emotions and a flexible application of regulatory strategies, leading to a more adaptive coping and a lower risk of somatization.^20,21,71,75,76^ Moreover, to comfortably sustain attention in bodily sensations, those must not be perceived as catastrophic, threatening, or misleading.^25,73^ Thus, IPV-targeted interventions must also consider the importance of restoring the safety and acquaintance of the survivor’s traumatized body.^78–80^

## Limitations and future directions

This study does not come out without limitations. The assessments were conducted in two distinct regions of Portugal, which limits the generalizability of the findings to other regions/countries. Additionally, differences in educational levels between the IPV and non-IPV groups may indicate variations in socioeconomic status (SES), potentially affecting overall health. Due to the temporary employment and household characteristics of the IPV group, socioeconomic data were not collected in this study, preventing control for this variable. Therefore, future research on the health and somatic complaints of IPV survivors should account for SES. Considering the cross-sectional nature of the casual relations between IPV, somatic complaints, and interoceptive abilities cannot be assumed. Therefore, future longitudinal studies will be helpful to gain a deeper understanding of our findings. Valuable research advances currently allow for a more robust and complex examination of the violence index, including not only types of violence but also its severity.^81,82^ Cross-cultural adaptation of such measures will be crucial to replicate the findings of the present study and enrich its discussion. Moreover, our study endorsed the importance of future studies examining the experience of violence in individuals reporting medically unexplained symptoms, especially concerning symptoms of hyperarousal such as hypervigilance, state anxiety, attention difficulties, sleep disturbances, and migraines. Furthermore, future research should consider the between individuals’ characteristics (besides between groups) regarding interoception attention regulation abilities and somatic complaints, as well as to extend these findings to men victims of IPV.

## Conclusion

The present findings provide insight into the contribution of interoception to the experience of somatic complaints by women who suffered IPV. Those women report significant somatic complaints consistent with a framework of heightened arousal. Furthermore, the ability to regulate the attention given to bodily sensations seems crucial for them to improve emotional and physiological regulation, leading to a lower risk of somatization and better health. Therapeutic interventions for survivors of IPV must consider promoting interoceptive attention regulation to help them use bodily sensations to self-regulate and manage health and illness symptoms adaptively.

## Supplemental Material

sj-docx-1-whe-10.1177_17455057251326013 – Supplemental material for Attention to the body! Comparing the connection between interoceptive abilities and somatic complaints of women with and without history of intimate partner violence Supplemental material, sj-docx-1-whe-10.1177_17455057251326013 for Attention to the body! Comparing the connection between interoceptive abilities and somatic complaints of women with and without history of intimate partner violence by Joana Machorrinho, José Marmeleira, Graça Duarte Santos and Guida Veiga in Women’s Health

## Declarations

**Ethics approval and consent to participate:** The study was approved by the University of Évora’s ethics committee (GD/31071/2018) and conducted in accordance with the Declaration of Helsinki (General Assembly of the World Medical Association, 2014) [(General Assembly of the World Medical Association 2014)^46^]. All participants provided written informed consent for participation in the study.

**Consent for publication:** All participants provided written informed consent for the publication of this study.

**Availability of data and materials:** The data are available from the authors upon reasonable request.
